# Acute deep neck infection MRI: deep learning segmentation and clinical relevance of retropharyngeal edema volume

**DOI:** 10.1186/s41747-026-00686-2

**Published:** 2026-02-23

**Authors:** Ville Sakari Viertonen, Aapo Sirén, Mikko Nyman, Heidi Huhtanen, Riku Klén, Jussi Hirvonen, Oona Rainio

**Affiliations:** 1 Department of Radiology, University of Turku and Turku University Hospital, Turku, Finland; 2 Turku PET Centre, University of Turku and Turku University Hospital, Turku, Finland; 3 Medical Imaging Centre, Department of Radiology, Tampere University and Tampere University Hospital, Tampere, Finland

**Keywords:** Artificial intelligence, Edema, Magnetic resonance imaging, Neural networks (computer), Respiratory tract infections

## Abstract

**Objective:**

Retropharyngeal edema (RPE) on MRI in patients with acute neck infection is associated with disease severity. We explored the potential role of RPE volume as a quantitative marker and developed a convolutional neural network (CNN) for automated RPE volume segmentation.

**Materials and methods:**

Volumes of RPE were manually segmented from T2-weighted fat-suppressed Dixon magnetic resonance (MR) images from 244 patients. These volumes were correlated with clinical variables, such as the need for intensive care unit (ICU) admissions, C-reactive protein (CRP) levels, maximal abscess diameter, and length of hospital stay (LOS). Manually segmented masks were used to train a CNN.

**Results:**

Patients who required ICU admission had significantly higher RPE volumes than those who did not, and RPE volume outperformed the binary RPE (presence/absence) in classification analysis of ICU admissions. Furthermore, RPE volume correlated positively with LOS, CRP, and maximal abscess diameter. At the slice level, the deep learning (DL)-based model achieved its highest area under the receiver operating characteristic curve (AUROC) in sagittal slices (98.2%) and its highest Dice similarity coefficient in axial slices (0.534).

**Conclusion:**

RPE volume is a promising quantitative imaging biomarker associated with relevant clinical outcomes in acute neck infections. Our DL-based model enables automated quantification of RPE volume.

**Relevance statement:**

RPE volume provides clinically meaningful information in acute neck infections, outperforming binary classification in predicting disease severity and correlating with key clinical outcomes. Automated DL-based segmentation accurately locates the RPE and provides a moderate quantitative measurement of RPE volume, supporting its potential as a clinical imaging biomarker.

**Key Points:**

- RPE volume correlated with markers of severe illness and outperformed binary RPE classification.
- We developed a DL-based algorithm for slice-wise classification and automatic segmentation of RPE.
- The classification model achieved excellent performance, while segmentation yielded modest Dice similarity coefficients consistent with prior imaging-based tumor segmentation algorithms.

**Graphical Abstract:**

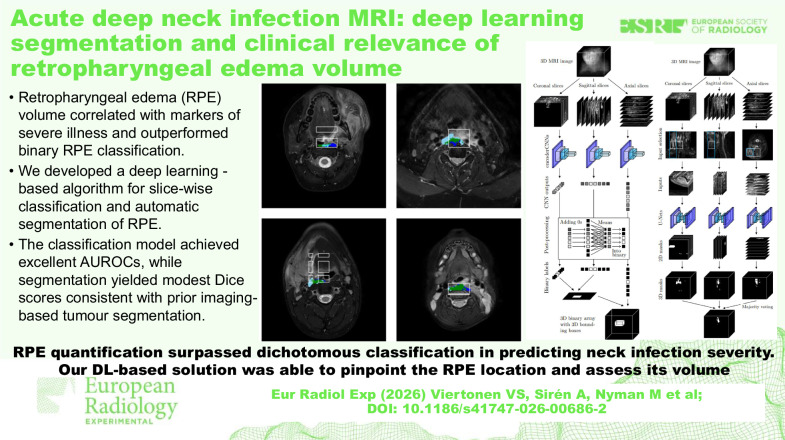

## Background

Acute deep neck infections present a clinical challenge even in tertiary care settings and often require multidisciplinary attention and invasive procedures. Clinical assessment of the deep cervical spaces is difficult, highlighting the critical role of imaging in the diagnostic process. Accurate diagnosis is essential, as treatment strategies, ranging from conservative management to surgical intervention, depend on factors such as the extent of the infection, the presence of surgically drainable abscesses, and complications [1,2]. Among imaging modalities, contrast-enhanced computed tomography is the most widely used due to its good availability and rapid acquisition times [3–6]. However, magnetic resonance imaging (MRI) has also been shown to be a feasible alternative even in acute settings, offering significant added value in detecting drainable abscesses with excellent accuracy [7]. Given the demonstrated advantages of MRI over computed tomography in acute settings, investigating its additional potential benefits is worthwhile.

Retropharyngeal edema (RPE) is a distinct reactive edema pattern located in the retropharyngeal space: a narrow potential compartment in the deep neck, bounded anteriorly by the buccopharyngeal fascia and posteriorly by the prevertebral fascia. On MRI, RPE is visualized as an area of high signal intensity on fat-suppressed T2-weighted sequences and can be identified by radiologists with substantial interobserver agreement [8]. The presence of RPE on a neck MRI is a clinically significant finding, associated with the need for intensive care unit (ICU) admissions [8]. In a large cohort, a multivariable analysis predicting ICU admissions revealed RPE as a notable individual predictor, alongside C-reactive protein (CRP) levels and maximal abscess diameter [9].

To assist radiologists in identifying RPE on MRI in an acute setting, Rainio et al [10] previously developed a deep learning (DL)-based automated detection method. The model was trained using axial T2-weighted fat-suppressed Dixon MRI from patients with acute neck infections, annotated by radiologists at both the patient and slice levels. Model performance was evaluated against expert radiologist assessments, using accuracy, sensitivity, specificity, and area under the receiver operating characteristic curve (AUROC) as metrics. The model reached an AUC of 94.1% at the slice level and 94.8% at the patient level, consistent with high discriminating performance [10].

In previous analyses, RPE has been considered a binary variable; the patient either has it or does not. However, we hypothesize that RPE might also contain quantitative information, specifically, that the extent (volume) of RPE could correlate more precisely with clinical outcome variables and thus offer more granular information. Therefore, this study aimed to: (1) test this hypothesis by correlating manually delineated RPE volumes with pertinent clinical and laboratory variables; and (2) develop a DL-based method for automatically segmenting RPE from T2-weighted fat-suppressed Dixon MRI.

## Materials and methods

### Patients and image data

We retrospectively reviewed patient data from a single academic tertiary care referral center as previously described [8,9], including patients with clinically confirmed acute neck infections who underwent emergency neck MRI between April 1, 2013, and August 30, 2021. Due to the study’s retrospective nature, no written patient consent or institutional review board review was obtained, as these are not required by the national legislature for retrospective studies of existing data. Details regarding the imaging equipment and protocols have been previously described [8]. Briefly, the T2-weighted fat-suppressed Dixon images used for RPE segmentation were acquired in the emergency radiology department, using a Philips Ingenia 3 T system equipped with a dS HeadNeckSpine coil (Philips Healthcare).

MRI consisted of 26‒60 axial slices whose size varied from 288 × 288 pixels to 640 × 640 pixels. The voxel width and height varied from 0.375 mm to 0.694 mm with a mean value ± standard deviation of 0.528 ± 0.149 mm. The voxel depth (the spacing between axial slices) ranged from 3.98 mm to 5.96 mm, with a mean ± standard deviation of 4.80 ± 0.228 mm.

### Software and hardware

Manual image segmentation was performed using ITK-SNAP (version 4.2.2) [11]. Analyses were done using R (version 4.4.0) [12] and the package *pROC* [13]. Intra- and interobserver reliability analyses were done using R and the *lme4* package. Other statistical analyses were done using IBM SPSS Statistics for Mac (version 28, IBM Corp., 2021). The convolutional neural network (CNN) models were built and trained with Python (version: 3.12.8) [14] by using the packages Tensorflow (version: 2.18.0) [15], Keras (version: 3.7.0) [16], and SciPy (version: 1.14.1) [17]. To estimate the computational feasibility of the models, all the CNNs were trained on the same computer with an Intel Core 5 125H processor and 16GB of random-access memory, and the time required by the experiments was recorded.

### Manual image segmentation

RPE was manually segmented slice-by-slice using ITK-SNAP, a third-party, open-source, multi-platform software application [11], followed by automated volume calculation (mm^3^). The area in which the high-intensity T2-signal RPE was segmented was defined by the following criteria: anteriorly by the posterior edge of the inferior constrictor muscle, laterally by the lateral margin of the inferior constrictor muscle and the anteromedial edge of the carotid sheath, and posteriorly by the anterior edge of the longus colli and scalene muscles. Segmentation extended from the level of the nasopharynx superiorly to the level of the C7–T1 vertebrae inferiorly. We only considered one continuous area of high signal per patient and disregarded any small secondary strands if they occurred. This aimed to optimize the conditions for the DL algorithm to reliably and consistently recognize patterns in the patient image material. Segmentations were performed by V.V. (Reader 1) under the supervision of two neuroradiologists (J.H. and A.S.). To measure intra- and interobserver agreement, a random sample of 50 patients was also blindly evaluated by a fellowship-trained neuro- and head & neck radiologist (J.H., Reader 2) and neuroradiologist (A.S., Reader 3) twice, at least 1 week apart. The final segments used in the analysis were confirmed in consensus between Reader 1 and Reader 2. The numbers of positive slices and volume of positive segmentation in the resulting binary masks is represented in Supplementary Tables S1 and S2, respectively.

### Statistical analyses of tabular data

Associations between RPE volume and clinical and prognostic data were analyzed using the Mann–Whitney *U* test, Spearman’s ρ, and multivariable models. Pediatric patients were included in the training of the DL models, as the relative anatomic structures do not differ substantially from those of adult patients. Clinical correlations were performed exclusively on the adult patient subgroup (age 18 or older) to avoid potential bias. The exclusion of pediatric patients from the clinical correlations was based on the difference in the volumetric scale of the anatomy, resulting in significantly smaller average RPE volumes. RPE volume was set to zero in patients without RPE. In univariate analyses, the associations between RPE volume and the need for intensive care, as well as the presence of abscesses, were assessed using the Mann–Whitney *U* test. Spearman’s ρ was used to examine the associations between RPE volume and length of hospital stay (LOS), CRP levels, and the maximum diameter of abscesses. Given that RPE was a significant binary predictor of ICU admission in the previous study, a multivariable model was built here for the prediction of ICU admission, using a logistic regression model with RPE volume, CRP, and the maximum diameter of abscesses as predictors. These predictors were selected *a priori* based on prior results [8,9]. ICU admission classification performance was also assessed with areas under the curve (AUROC), and compared between binary RPE and RPE volume using DeLong’s test.

### Reliability assessment

Intraobserver and interobserver agreement were assessed using the intraclass correlation coefficient (ICC) based on two-way mixed-effects models for absolute agreement. The ICC represents the proportion of total variance attributable to between-subject (interindividual) variance relative to within-subject (interobserver or intraobserver) variance, and was interpreted as follows: < 0.5, poor; 0.5–0.75, moderate; 0.75–0.9, good; > 0.9, excellent. For intraobserver reliability, ICC(1,1) values were calculated separately for each reader using one-way random-effects models (treating sessions as replicates within patients). For interobserver reliability, ICC(2,1) was estimated using a two-way mixed-effects model on session-averaged values per reader, accounting for repeated observations within readers and assuming fixed reader effects. 95% confidence intervals were approximated via Fisher z-transformation.

### Convolutional neural networks

**Fig. 1:**
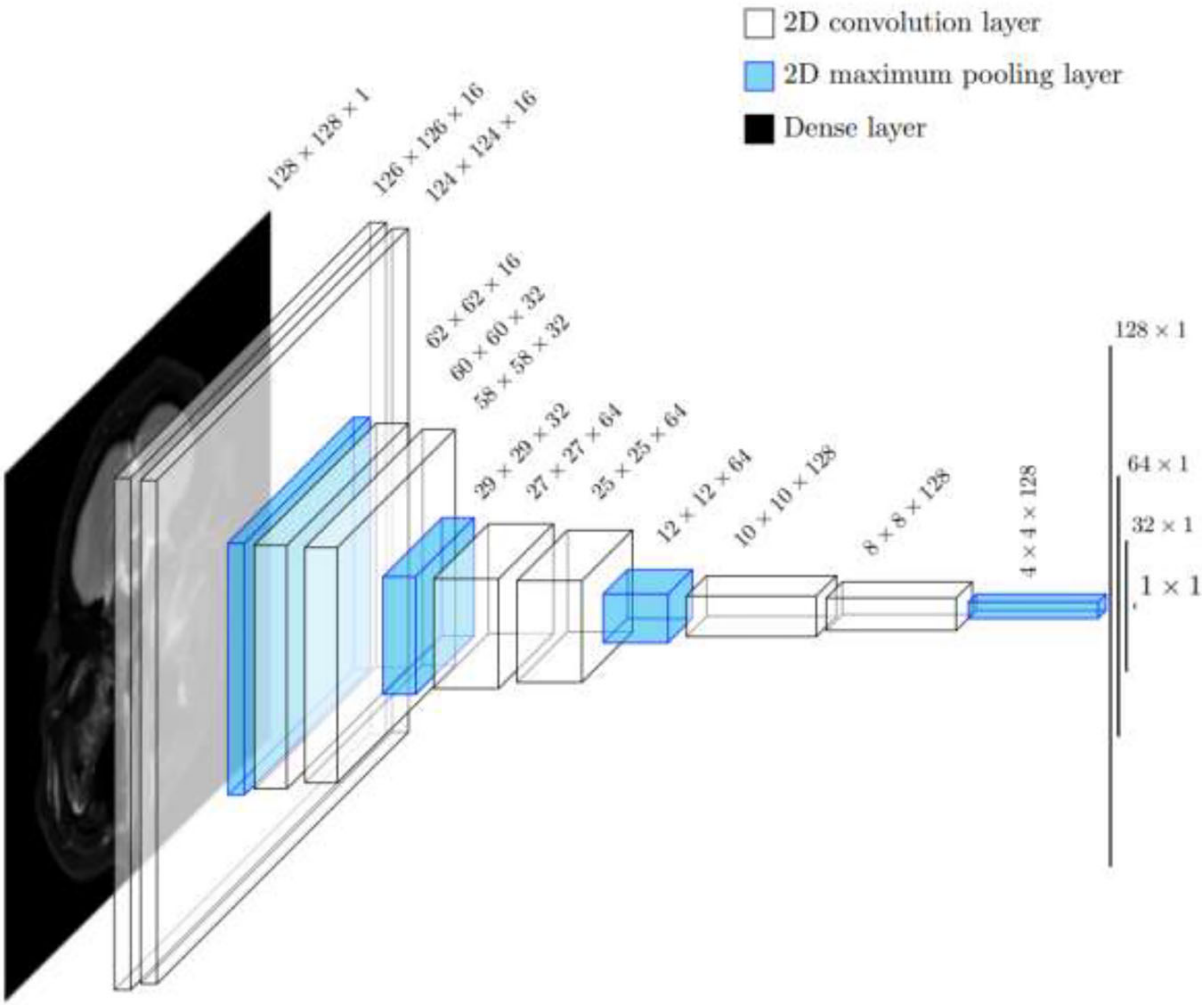
The architecture of encoderCNN used for classification. The CNN receives an input of 128 × 128 × 1 elements and returns a single numeric value as an output, and this value can be converted into a binary label with a suitable threshold. The total number of parameters in the CNN is 565,873. CNN, Convolutional neural network

In this study, we used two different CNN designs, both of which were inspired by the U-Net architecture introduced in 2015 by Ronneberger et al [18]. For classification, we used a recently introduced CNN from Hellström et al [19], referred here as encoderCNN. As illustrated in Fig. 1, it consists of four sequences of paired convolutions and maximum pooling operations, like in U-Net’s encoder, followed by four dense layers. As noted in earlier [10,19], this CNN enables accurate yet computationally very fast classification of medical images. In our experiments, encoderCNN was trained with binary cross-entropy as a loss function and Adam with a learning rate of 0.001 as an optimizer for five epochs.

**Fig. 2:**
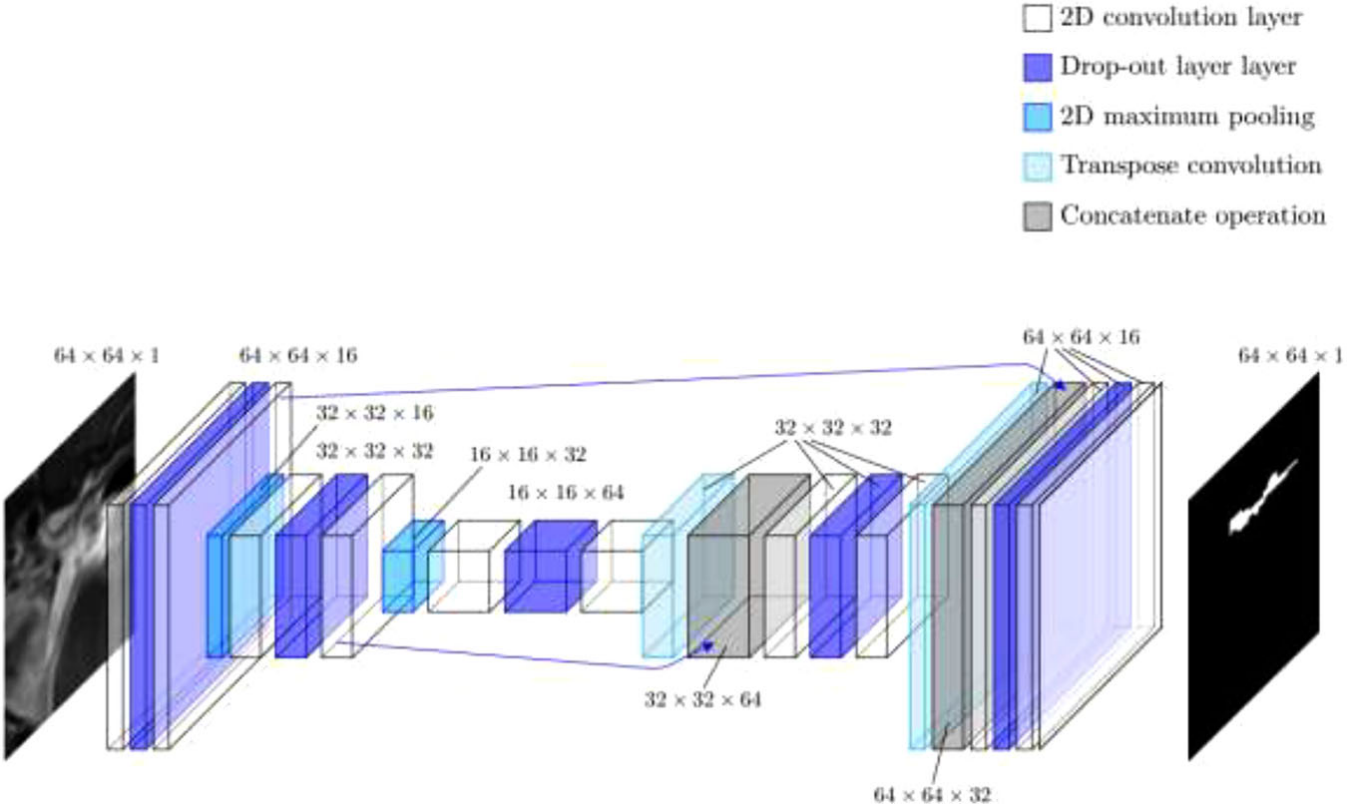
The architecture of the U-Net-type segmentation CNN. When receiving an input of 64 × 64 × 1 elements, the CNN returns a binary mask of the same size. The output of two convolution layers in the encoder is copied and given to the concatenation layers of the decoder, as marked with blue arrows. The total number of parameters in this CNN is 116,753. CNN, Convolutional neural network

For segmentation, we used the typical U-Net CNN modified to suit images of 64*64 pixels, as shown in Fig. 2. Namely, U-Net is the most popular segmentation CNN for medical images in particular due to its high accuracy even with limited amounts of training data and computational resources [19]. Our U-Net was trained with the Dice loss function and the optimizer Adam with a learning rate of 0.001 for 30 epochs.

### Structure of our experiment

The positive patients were randomly divided into five groups with five-fold cross-validation, and the whole process was repeated five times for the resulting training and test sets so that the same images are unseen for both classification and segmentation CNNs.

**Fig. 3:**
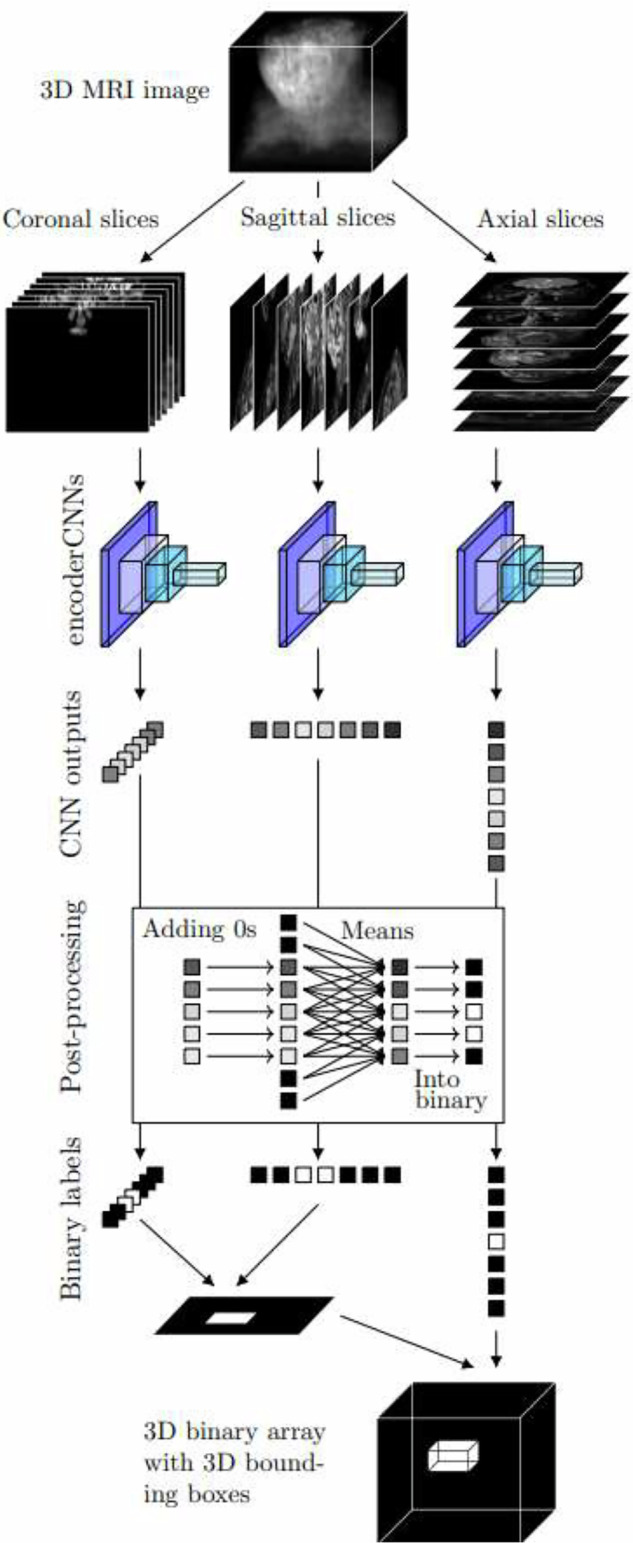
The process of creating three-dimensional (3D) bounding boxes based on slice-wise classification with three encoder CNNs classifying coronal, sagittal, and axial slices

For classification, we extracted all the coronal, sagittal, and axial slices from the original image volumes and scaled them to a size of 128 × 128 pixels, the values of which were converted into float16 numbers on the interval (0,1). By using all the pre-processed slices of our training data, we trained three separate versions of encoderCNN to classify coronal, sagittal, and axial slices based on whether there was positive segmentation on the given slice according to its binary mask. Due to the five-fold cross-validation, the exact size of the training and test set data varied, as shown in Supplementary Table S3. After the training, we predicted all the pre-processed coronal, sagittal, and axial slices for both the training and the test sets. To post-process the outputs, we used the moving average technique described in [10] and converted the numeric outputs into binary labels by using the maximal Youden’s threshold of the training set predictions. The process from slice-wise classification to the creation of three-dimensional (3D) bounding boxes is described in Fig. 3: the predicted binary labels were used to predict 3D bounding boxes for the RPE segmentation by assuming that a voxel was positive if and only if it lay in the intersection of coronal, sagittal, and axial slices predicted as positive.

The predicted bounding boxes were then used to sample data for the segmentation: By using strides of 32 pixels, we chose such squares of 64 × 64 pixels that contained either at least one third of the predicted bounding box surface of the original coronal, sagittal, or axial slice or at least 1,000 voxels within the predicted bounding box. To obtain squares of 64 × 64 pixels, stretching was used for sagittal and coronal slices of 3D images with fewer than 64 axial slices. All the resulting squares were pre-processed by scaling their pixel values into float16 elements on the interval (0,1). For the squares sampled from the training data, we also collected the ground-truth RPE segmentation masks to obtain suitable ground-truth two-dimensional (2D) masks of 64 × 64 pixels.

**Fig. 4:**
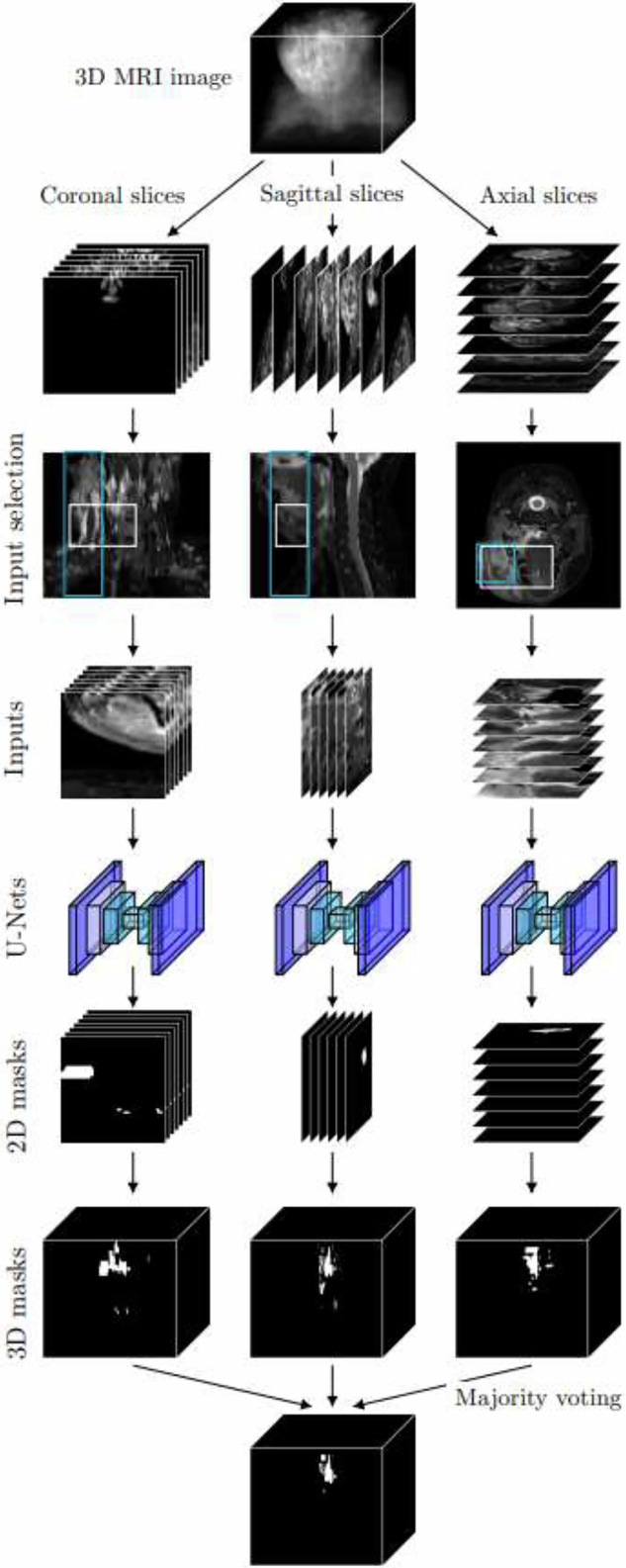
The process of predicting segmentation masks with U-Nets performing the segmentation for coronal, sagittal, and axial input slices. The inputs are selected by using information from the three-dimensional (3D) bounding boxes created as in Fig. 3

We trained three different U-Nets to perform segmentation separately for coronal, sagittal, and axial slices. The number of slices in the training sets is presented in Supplementary Table S3. For the patients in the test dataset, we first initialized three 3D zero arrays of the size of the patient’s MRI image, predicted squares collected in the same way as for the training set, and inserted the predicted segmentation results into the location of the squares within the 3D image space. We created separate masks from the coronal, sagittal, and axial square selections. We also obtained a fourth 3D segmentation mask via majority voting from the three earlier masks, a method referred to here as the 2.5-dimensional segmentation, as opposed to coronal, sagittal, or axial segmentation. The whole process of obtaining the segmentation masks is described in Fig. 4.

### Evaluation of DL models

In a binary classification task, each prediction is either true positive (TP), false negative (FN), false positive (FP), or true negative (TN). By counting the numbers of TP, FN, FP, and TN observations, we can define four evaluation metrics:

$${{{\rm{Accuracy}}}}=\frac{{TP}+{TN}}{{TP}+{FN}+{TN}}$$

$${{{\rm{Sensitivity}}}}=\frac{{TP}}{{TP}+{FN}}$$

$${{{\rm{Specificity}}}}=\frac{{TN}}{{TN}+{FP}}$$

$${{{\rm{Precision}}}}=\frac{{TP}}{{TP}+{FP}}$$

In addition to these metrics, we used the AUROC as a fifth evaluation metric for classification. To evaluate the results of segmentation, we calculated the Dice similarity score as

$${{{\rm{Dice}}}}=\frac{2\cdot \left|X\cap {{{\rm{Y}}}}\right|}{\left|X\right|+\left|Y\right|}$$

where |X| is the number of positive voxels in the predicted segmentation mask, |Y| is the number of positive voxels in the ground-truth segmentation mask, and |X ∩ Y| is the number of voxels in the intersection of the voxels predicted as positive and the ground-truth positive voxels.

During the evaluation, we computed the values of accuracy, sensitivity, specificity, precision, and AUROC for the coronal, sagittal, and axial slice predictions separately for each patient over all the test sets. The Dice similarity coefficients were similarly computed for all the 3D masks. To estimate whether there were statistically significant differences in patient-wise values of different evaluation metrics between coronal, sagittal, and axial views, we used the Wilcoxon signed-rank test with a default significance level of 0.05. Pearson’s correlation, alongside the related *t*-test of significance of correlation, was used to estimate the potential correlation between the real RPE volume and the resulting Dice similarity coefficients. Additionally, the predicted RPE volume was compared to real RPE volume in terms of Pearson’s correlation, mean absolute error, and mean relative error.

## Results

### Interobserver agreement of RPE volume

The average intrarater ICC was 0.992 (95% confidence interval 0.985–0.995), indicating excellent intraobserver agreement. The model-based interobserver ICC was 0.827 (95% confidence interval 0.768–0.872), indicating good interobserver agreement.

### Clinical associations

**Fig. 5:**
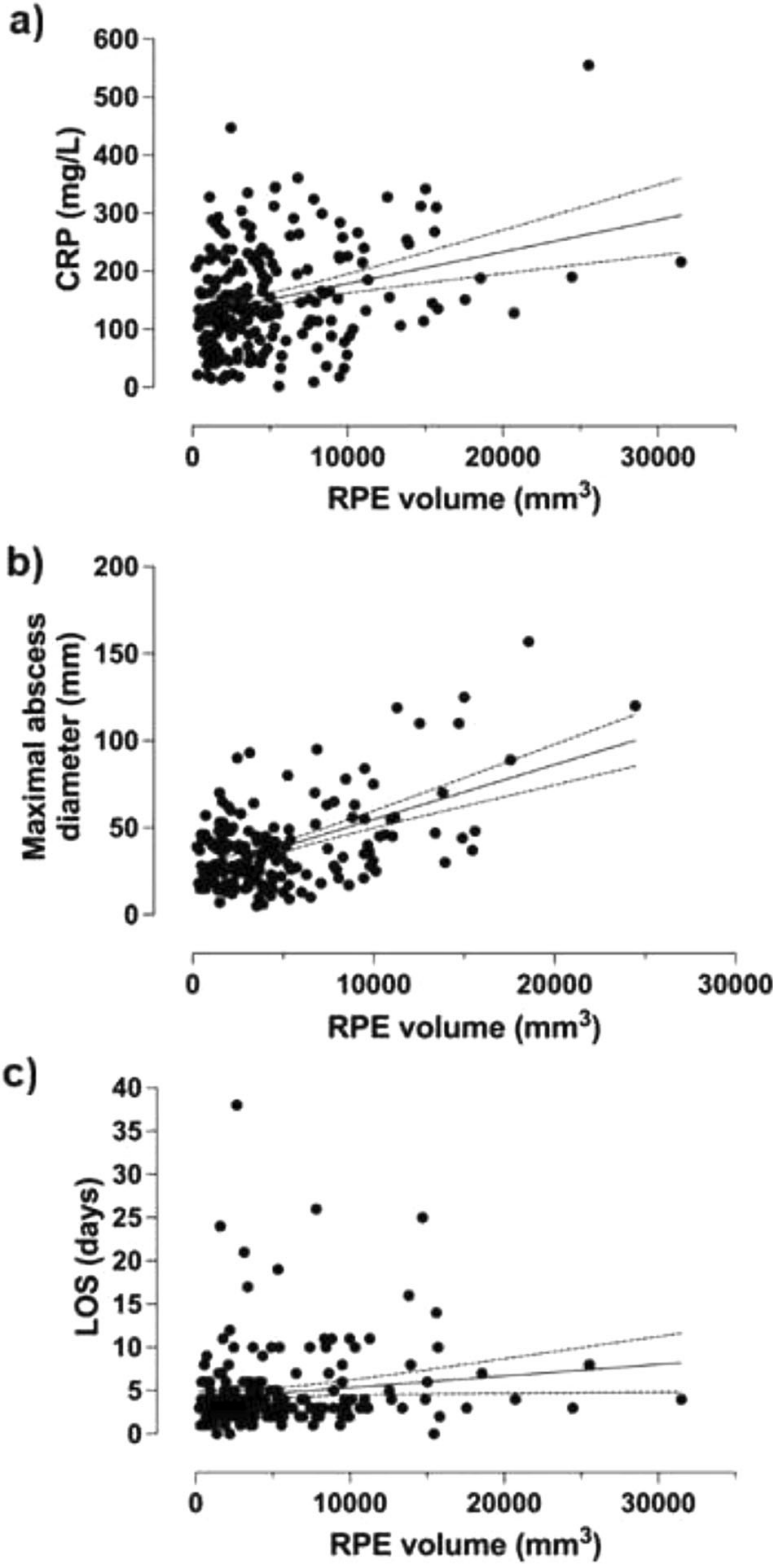
Correlations between retropharyngeal edema (RPE) volume and clinical parameters in adult patients. Scatter plots demonstrate positive associations of RPE volume with (**a**) C-reactive protein (CRP), (**b**) maximal abscess diameter, and (**c**) length of hospital stay (LOS). Solid lines represent regression lines, and dashed lines represent the 95% confidence intervals. Zeros were omitted for visual purposes

The study included 479 patients, divided into 422 adults (mean age, 46 years; 58% male, 42% female) and 57 children (12% of the total). Among the adult patients, 217 (51%) were RPE-positive and 205 (49%) were RPE-negative. The mean RPE volume among RPE-positive adult patients was 5,028 mm³, with a median of 3,554 mm³. In adult patients, the volumetric extent of RPE ranged from 234 to 31,490 mm³. Adult patients requiring ICU admission had, on average, higher RPE volumes (6,003 mm³) than those who did not (2,157 mm³) (*p* = 0.004). In addition, RPE volume was positively correlated with LOS (*p* = 0.039), CRP (*p* < 0.001), and greater maximal abscess diameter (*p* = 0.010), as shown in Fig. 5. However, average RPE volume was similar in adult patients with abscess (2,812 mm³) and those without (2,069 mm³) (*p* = 0.904).

In the multivariable logistic regression model predicting ICU admissions, RPE volume showed a trend toward statistical significance (*p* = 0.086), while maximal abscess diameter (*p* = 0.001) and CRP (*p* < 0.001) were statistically significant.

In the ICU classification analysis, AUROCs and their 95% confidence intervals were as follows: RPE 0.714 (0.665–0.764), RPE volume 0.775 (0.710–0.841), CRP 0.764 (0.682–0.845), and maximal abscess diameter 0.742 (0.650–0.834). The optimal cutoff for RPE volume, determined by Youden’s index, was 1,389 mm^3^, with a sensitivity of 0.889 and specificity of 0.618. A DeLong’s test indicated that RPE volume significantly outperformed binary RPE classification in discrimination performance (*p* = 0.004).

### Deep learning models

The patient-wise accuracy, sensitivity, specificity, precision, and AUROC values for classifying the coronal, sagittal, and axial slices based on the presence of RPE in all 244 positive patients are summarized in Table 1. Based on Wilcoxon signed-rank tests for patient-wise AUROC values, the classification performance was significantly better for sagittal slices than for coronal slices (*p* = 0.00585), but it did not differ between coronal and axial slices (*p* = 0.506) nor sagittal and axial slices (*p* = 0.654). The average training times varied from 3.6 min to 129.5 min for classification and segmentation models, as shown in Supplementary Table S4.

**Table 1:** Mean ± standard deviation of patient-wise accuracy, sensitivity, specificity, precision, and area under the receiver operating characteristic curve (AUROC) values obtained by computing the values of these evaluation metrics by classifying coronal, sagittal, and axial slices for each patient in each of the five test sets

View	Accuracy	Sensitivity	Specificity	Precision	AUROC
Coronal	0.944 ± 0.040	0.802 ± 0.220	0.959 ± 0.041	0.679 ± 0.254	0.974 ± 0.039
Sagittal	0.930 ± 0.041	0.860 ± 0.169	0.948 ± 0.047	0.773 ± 0.197	0.982 ± 0.023
Axial	0.891 ± 0.065	0.889 ± 0.198	0.894 ± 0.089	0.749 ± 0.229	0.971 ± 0.056

**Fig. 6:**
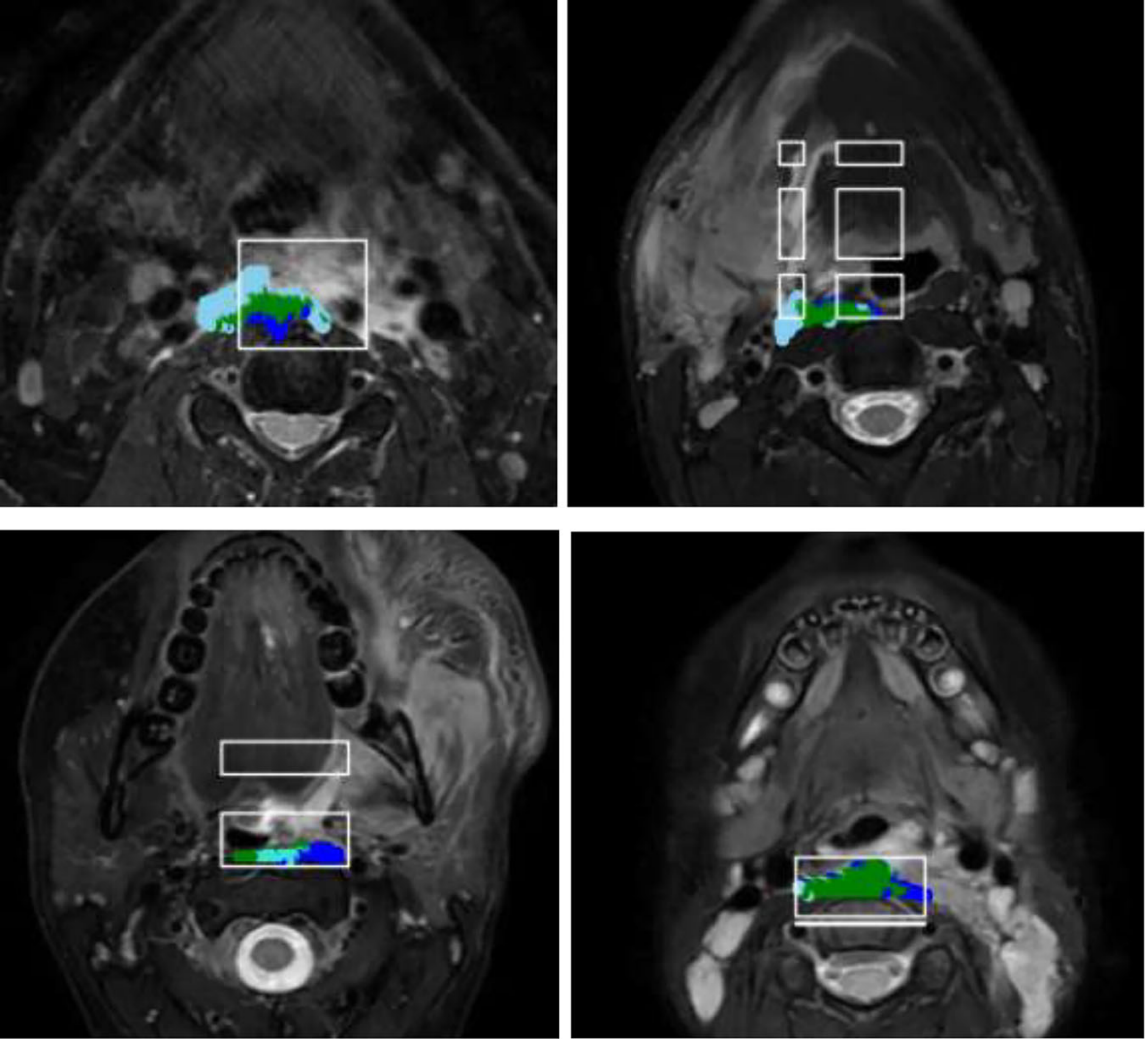
Examples of predicted segmentation results obtained with the axial segmentation model. The true-positive areas are denoted by green color, the false-negative areas by light blue color, the false areas by blue color, and the bounding box outlined by white color

The patient-wise Dice similarity coefficients are presented in Table 2. According to these Dice similarity coefficients, the axial segmentation model worked the best, though the sagittal model produced the smallest number of fully wrong segmentation predictions with a Dice similarity coefficient of 0. Based on Wilcoxon signed-rank tests, the axial segmentation model resulted in significantly higher Dice similarity coefficients than the coronal segmentation model (*p* < 0.001) or the sagittal segmentation model (*p* < 0.001), but there was no statistically significant difference between the axial segmentation model and the 2.5-dimensional approach (*p* = 0.392). Four example cases of the segmentation results are visualized in Fig. 6.

**Table 2:** The mean ± standard deviation (SD), minimum, median, and maximum values for the patient-wise Dice similarity coefficients obtained by computing the Dice similarity coefficients from the coronal, sagittal, axial, and 2.5-dimensional (2.5D) segmentation masks for each patient in each of the five test sets

View	Mean ± SD	Minimum	Median	Maximum
Coronal	0.39 ± 0.21	0 (for 12 patients)	0.424	0.781
Sagittal	0.464 ± 0.192	0 (for 6 patients)	0.490	0.808
Axial	0.534 ± 0.195	0 (for 11 patients)	0.576	0.836
2.5D approach	0.518 ± 0.208	0 (for 8 patients)	0.567	0.843

There was a low but statistically significant correlation between the Dice similarity coefficients obtained with the axial segmentation model and the real RPE volume according to the ground-truth binary masks (Pearson’s correlation: 0.216, *p* < 0.001). All the 11 patients for whom the axial segmentation model produced a Dice similarity coefficient of 0 had a true RPE volume less than 330 mm^3^. For the 132 patients with a real RPE volume of at least 3,000 mm^3^, the axial segmentation model produced Dice similarity coefficients with a mean ± standard deviation of 0.596 ± 0.152 and a median of 0.621.

**Fig. 7:**
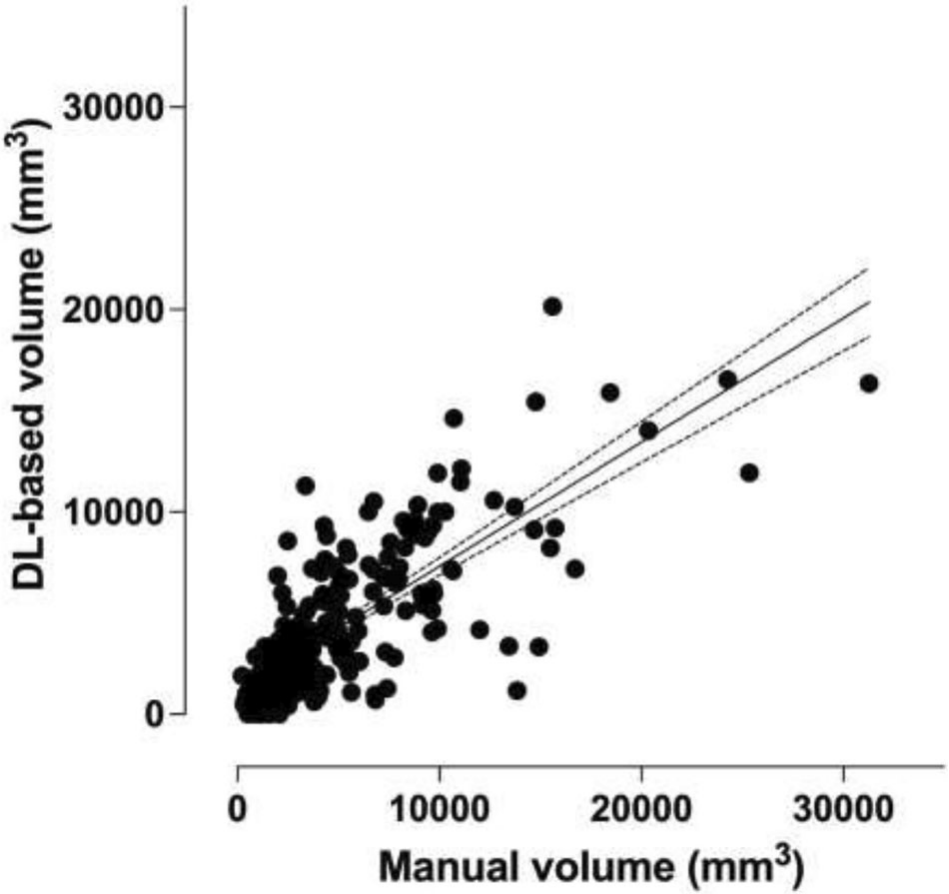
Correlations between the real and the predicted retropharyngeal edema (RPE) volume

There was a high and statistically significant correlation with a Pearson’s correlation coefficient of 0.775 (*p* < 0.001) between the real RPE volumes and the predicted RPE volumes computed based on the axial segmentation model. This relationship is depicted in Fig. 7. The related mean absolute error was 1,810 mm^3^, and the mean relative error was 48.9%.

## Discussion

RPE is a reactive edema pattern associated with a severe course of deep neck infection. We explored the clinical significance of RPE volume in acute deep neck infection patients by utilizing fat-suppressed T2-weighted MRI and developed a DL-based algorithm for its automated segmentation.

Automated segmentation was a challenging task due to the limited amount of data and the small size of the ground-truth positive RPE areas with respect to the background. The proposed DL approach first performed slice-wise classification to obtain an initial guess of the RPE location in the 3D image and then utilized the resulting 3D bounding box prediction to select smaller areas of interest for segmentation. In clinical correlation studies, we show that RPE volume is associated with markers of severe illness and outperforms binary RPE classification.

The slice-wise classification model was an extension of our previous work on RPE classification by DL. The mean AUROC in axial slices was 97.1%, compared with 94.1% in the previous study [10] due to minor methodological differences. Based on our comparison between the different views, sagittal slices yielded the best AUROC, though the sensitivity was highest for axial slices. The mean accuracy was between 89% and 95% for all three views.

The 3D bounding boxes obtained via slice-wise classification were necessary for the segmentation. As can be seen from Supplementary Table S2, the amount of RPE was very small relative to the total image area, posing significant challenges for accurate segmentation. The scarcity of positive pixels (between 0.0023–0.31% of the whole 3D image) prevented the convergence of the segmentation CNN on uncropped slices without a bounding box approach, even in combination with the Dice loss function that does not reward TN segmentation. The 3D bounding boxes were not directly suited for the prediction of RPE volume by themselves because only a small part (from 0.022% to 37%, with a median value of 4.1%) of the total bounding box volume was positive RPE volume, leading to a mean Dice similarity coefficient of 0.091 ± 0.065 between the real RPE volume and the predicted 3D bounding boxes. However, the bounding box approach allowed us to select smaller inputs for the segmentation CNN so that we can focus the CNN’s attention on the regions of interest specifically and exclude large amounts (over 99.8%) of negative image background. The technical benefit of this approach was also a decreased amount of computational resources, such as time and memory.

Despite highly accurate classification, the segmentation results were modest in terms of Dice. We found that the axial plane achieved the highest Dice similarity coefficient (0.534), followed by the 2.5-dimensional approach (0.518). The reason segmentation performance was poorer on coronal and sagittal models might be that the selection of inputs of 64 × 64 pixels required scaling images due to the varying axial length of the original 3D images. Potentially, if all the 3D images had the same dimensions, the 2.5-dimensional approach could have been more useful. Additionally, considering that our segmentation task is about locating small and irregularly-shaped, heterogeneous targets, the current performance was expected: The Dice similarity coefficients slightly over 0.50 are comparable to those previously reported for MRI-based segmentation of prostate cancer [20–22] and head and neck cancer tumors [23]. While very small edema volumes may have less prognostic or clinical relevance compared to larger ones when assessing the impact of RPE, their inclusion in model training remains important for comprehensive performance evaluation. Including all applicable cases also enhances the model’s generalizability, ensuring that its performance extends reliably across the full spectrum of edema volume presentations in the population.

A DL-based algorithm could present a clinical advantage, as accurate estimation of RPE volumes may provide clinically meaningful information. Our findings of larger RPE volumes correlating with a more severe disease phenotype in adult patients validate our hypothesis of RPE being a useful quantitative biomarker. Clinically, segmented RPE volumes correlated with ICU admissions, surpassing binary RPE classification, and were positively associated with CRP and LOS. These findings confirm that RPE volumes offer more granular data for patient prognostication, encouraging the exploration of quantitative MRI biomarkers to enhance clinical relevance in emergency radiology and support refined risk stratification. We found an optimal volume cutoff of 1,389 mm^3^. Based on this, a threshold RPE volume could be integrated into an acute patient care setting and might help flag patients at higher risk who warrant closer follow-up. However, this cutoff should be validated in larger, longitudinal studies before clinical implementation. While time-consuming to manually segment at the emergency department, automated methods might help in clinical deployment. It is worth emphasizing that the RPE represents reactive edema and not a surgically drainable collection, and that the exact pathophysiology of RPE in acute neck infections is yet to be elucidated.

This study further refines previous research demonstrating the clinical significance of binary RPE classification by Heikkinen et al [8] and Vierula et al [9] and the feasibility of DL models in RPE recognition by Rainio et al [10]. The strengths of this study are similar to the studies cited: a large sample size of clinically validated high-quality neck infection MRI images and good technical model performance in terms of training times for classification. A notable strength of this study is the precise definition of RPE, established through clearly delineated anatomical landmarks, and corroborated by excellent interobserver agreement. Nonetheless, the interpretation of RPE patterns remains inherently subjective. In terms of other limitations, the data used in manual image segmentation and training of the DL model were retrospectively acquired from only a single institution. We did not have an external test set available, so only five-fold cross-validation could be used, limiting the ability to assess generalizability across independent cohorts and necessitating further validation in future external datasets and a prospective validation after deployment into clinical workflows. Regarding improvements in model performance, training with a larger multi-institutional dataset and utilizing advanced data augmentation or 3D convolutional approaches may enhance segmentation accuracy. In addition, prospective multi-center studies in the future would increase the robustness of this study, as model performance may vary across different institutions, scanners, and sequence parameters.

In conclusion, volumetric quantification of RPE surpassed binary classification in predicting disease severity. The proposed DL-based solution was able to pinpoint the location of the RPE on MRI and assess the volume with moderate accuracy. With future improvements in segmentation accuracy, larger datasets, and external validation, our DL method could be improved further for more accurate prediction of RPE volume. These findings support RPE volume as a promising quantitative imaging biomarker.

## Supplementary information

ELECTRONIC SUPPLEMENTARY MATERIAL

## Data Availability

Data cannot be publicly shared because of the national legislatures on the privacy of patient data.

## Abbreviations

3D: Three-dimensional
AUROC: Area under the receiver operating characteristic curve
CNN: Convolutional neural network
CRP: C-reactive protein
DL: Deep learning
FN: False negatives
FP: False positives
ICC: Intraclass correlation coefficient
ICU: Intensive care unit
LOS: Length of hospital stay
MRI: Magnetic resonance imaging
RPE: Retropharyngeal edema
TN: True negatives
TP: True positives

## Code availability

The code is available at https://github.com/vviertonen/Acute-neck-infections/blob/main/rpeSeg.ipynb.

## References

[1] Asairinachan A, Santucci W, Kwok MMK et al (2025) Management of deep neck space infections—an Australian otolaryngology experience. ANZ J Surg 95:727–732. 10.1111/ans.19396 39812241 10.1111/ans.19396

[2] Sheikh Z, Yu B, Heywood E et al (2023) The assessment and management of deep neck space infections in adults: a systematic review and qualitative evidence synthesis. Clin Otolaryngol 48:540–562. 10.1111/coa.14064 37147934 10.1111/coa.14064

[3] Caprioli S, Tagliafico A, Fiannacca M et al (2022) Imaging assessment of deep neck spaces infections: an anatomical approach. Radiol Med 128:81–92. 10.1007/s11547-022-01572-8 36574110 10.1007/s11547-022-01572-8

[4] Maroldi R, Farina D, Ravanelli M et al (2012) Emergency imaging assessment of deep neck space infections. Semin Ultrasound CT MRI 33:432–442. 10.1053/j.sult.2012.06.008 10.1053/j.sult.2012.06.008 22964409

[5] Hagelberg J, Pape B, Heikkinen J et al (2022) Diagnostic accuracy of contrast-enhanced CT for neck abscesses: a systematic review and meta-analysis of positive predictive value. PLoS One 17:e0276544. 10.1371/journal.pone.0276544 36288374 10.1371/journal.pone.0276544 PMC9604924

[6] Hirvonen J, Lingam RK, Connor S (2025) ESR Essentials: acute infections of the head and neck—practice recommendations by the European Society of Head and Neck Radiology. Eur Radiol 36:334–343. 10.1007/s00330-025-11818-4 40702317 10.1007/s00330-025-11818-4 PMC12712113

[7] Nurminen J, Velhonoja J, Heikkinen J et al (2021) Emergency neck MRI: feasibility and diagnostic accuracy in cases of neck infection. Acta Radiol 62:735–742. 10.1177/0284185120940242 32660316 10.1177/0284185120940242 PMC8167911

[8] Heikkinen J, Nurminen J, Velhonoja J et al (2022) Clinical and prognostic significance of emergency MRI findings in neck infections. Eur Radiol 32:1078–1086. 10.1007/s00330-021-08200-5 34331114 10.1007/s00330-021-08200-5 PMC8794929

[9] Vierula J-P, Merisaari H, Heikkinen J et al (2025) MRI-based risk factors for intensive care unit admissions in acute neck infections. Eur J Radiol Open 14:100648. 10.1016/j.ejro.2025.100648 40236980 10.1016/j.ejro.2025.100648 PMC11999491

[10] Rainio O, Huhtanen H, Vierula J-P et al (2025) Deep learning detects retropharyngeal edema on MRI in patients with acute neck infections. Eur Radiol Exp. 10.1186/s41747-025-00599-6 10.1186/s41747-025-00599-6 PMC12179047 40536731

[11] Yushkevich PA, Piven J, Hazlett HC et al (2006) User-guided 3D active contour segmentation of anatomical structures: significantly improved efficiency and reliability. Neuroimage 31:1116–1128. 10.1016/j.neuroimage.2006.01.015 16545965 10.1016/j.neuroimage.2006.01.015

[12] R Core Team (2025) R: a language and environment for statistical computing. Version 4.5.2 (2025-10-31). Available via https://cran.r-project.org/doc/manuals/r-release/fullrefman.pdf

[13] Robin X, Turck N, Hainard A et al (2011) pROC: an open-source package for R and S+ to analyze and compare ROC curves. BMC Bioinformatics 12:77. 10.1186/1471-2105-12-77 21414208 10.1186/1471-2105-12-77 PMC3068975

[14] Van Rossum G, Drake F (2009) Python 3 reference manual. CreateSpace, Scotts Valley

[15] Abadi M, Agarwal A, Barham P et al (2015) TensorFlow: Large-Scale Machine Learning on Heterogeneous Systems. Available online: https://www.tensorflow.org/ (accessed on 14 September 2025)

[16] Chollet F (2015) Keras. GitHub https://github.com/keras-team/keras.git

[17] Virtanen P, Gommers R, Oliphant TE et al (2020) SciPy 1.0: fundamental algorithms for scientific computing in Python. Nat Methods 17:261–272. 10.1038/s41592-019-0686-2 32015543 10.1038/s41592-019-0686-2 PMC7056644

[18] Ronneberger O, Fischer P, Brox T (2015) U-Net: convolutional networks for biomedical image segmentation. In: Navab N, Hornegger J, Wells WM, Frangi AF (eds) Medical image computing and computer-assisted intervention, Medical Image Computing and Computer-Assisted Intervention – MICCAI 2015. Lecture Notes in Computer Science, vol 9351. Springer, Cham. 10.1007/978-3-319-24574-4_28

[19] Hellström H, Liedes J, Rainio O et al (2023) Classification of head and neck cancer from PET images using convolutional neural networks. Sci Rep 13:10528. 10.1038/s41598-023-37603-1 37386289 10.1038/s41598-023-37603-1 PMC10310830

[20] Hoar D, Lee PQ, Guida A et al (2021) Combined transfer learning and test-time augmentation improves convolutional neural network-based semantic segmentation of prostate cancer from multi-parametric MR images. Comput Methods Programs Biomed 210:106375. 10.1016/j.cmpb.2021.106375 34500139 10.1016/j.cmpb.2021.106375

[21] Lai C-C, Wang H-K, Wang F-N et al (2021) Autosegmentation of prostate zones and cancer regions from biparametric magnetic resonance images by using deep-learning-based neural networks. Sensors (Basel) 21:2709. 10.3390/s21082709 33921451 10.3390/s21082709 PMC8070192

[22] Simeth J, Jiang J, Nosov A et al (2023) Deep learning-based dominant index lesion segmentation for MR-guided radiation therapy of prostate cancer. Med Phys 50:4854–4870. 10.1002/mp.16320 36856092 10.1002/mp.16320 PMC11098147

[23] Schouten JPE, Noteboom S, Martens RM et al (2022) Automatic segmentation of head and neck primary tumors on MRI using a multi-view CNN. Cancer Imaging 22:8. 10.1186/s40644-022-00445-7 35033188 10.1186/s40644-022-00445-7 PMC8761340

