# Supplementary material for: Acute deep neck infection MRI: deep learning segmentation and clinical relevance of retropharyngeal edema volume
Source: Eur Radiol Exp. 2026 Feb 23;10:15. doi: 10.1186/s41747-026-00686-2 (PMC12929749; doi:10.1186/s41747-026-00686-2)
Supplement: Supplementary file 1 — ELECTRONIC SUPPLEMENTARY MATERIAL [file 41747_2026_686_MOESM1_ESM.pdf]

Acute deep neck infection MRI: deep learning segmentation and clinical relevance of retropharyngeal edema volume

ELECTRONIC SUPPLEMENTARY MATERIAL

Supplement table S1. The number of coronal, sagittal, and axial slices in the 244 MRI images, both in total and only for positive slices, and the percentage of positive slices among all the slices. A slice is considered positive if it contains at least one positive voxel according to the binary mask.

View	Slices in total	Positive slices	Percentage of positive slices
Coronal	100,400	9,321	9.28%
Sagittal	100,400	17,931	17.9%
Axial	11,197	3,378	30.2%

Supplement table S2. The mean ± SD, minimum, median, and maximum values for the numbers of positive voxels in the binary segmentation mask, the percentage of the positive voxels from the total size of the mask, and RPE volume per patient according to the binary segmentation masks.

	Mean ± SD	Minimum	Median	Maximum
Positive voxels in a mask	4720 ± 5800	158	2305	41276
Percentage of positive voxels in a binary mask	0.053 ± 0.049 %	0.0023%	0.038%	0.31%
RPE volume per patient (mL)	4.74 ± 4.55	0.230	3.37	31.3

Supplement table S3. The total number of slices and 64*64 squares from all the patients in a single training or test set created with five-fold cross-validation for our classification and segmentation tasks.

View	Classification task		Segmentation task	
	Slices in total for all the patients in a single training set	Slices in total for all the patients in a single test set	64*64 squares in total for all the patients in a single training set	64*64 squares in total for all the patients in a single training set
Coronal	75,952 – 84,992	15,408 – 24,448	22,096 – 24,368	4,511 – 7,787
Sagittal	75,952 – 84,992	15,408 – 24,448	34,642 – 38,940	8,311 – 14,662
Axial	8,457 – 9,509	1,688 – 2,740	22,641 – 24,898	4,252 – 7,651

Supplement table S4. The mean ± SD of the training times (in minutes) for the classification and segmentation models over the five different iterations of the five-fold cross-validation. The time to train the 2.5D approach for a specific iteration round is calculated as the sum of the times to train both coronal, sagittal, and axial models.

View	Classification model	Segmentation model
Coronal	29.9 ± 2.0	55.5 ± 3.4
Sagittal	30.2 ± 1.5	129.5 ± 10.1
Axial	3.6 ± 0.5	84.9 ± 6.8
2.5D approach	-	269.9 ± 17.8

Supplement table S5. Continuous variables in RPE-positive patients.

With RPE	Mean ± SD	Mamimum	Median	Minimum
Age (years)	43,6 ± 21.5	88	43	0
RPE volume (mm^3)	4848 ± 4676	31490	3465	234
Hospital stay lenght (days)	5 ± 4.57	38	3	0
CRP (mg/l)	146 ± 88.6	555	131	2
Maximal abscess diameter (if present, mm)	37.5 ± 24.0	157	27	5

Supplement table S6. Demographics and binary variables in RPE-positive patients.

With RPE		
Sex (male/female)	151 (61.9 %)	93 (38.1 %)
Adult/Pediatric	217 (88.9 %)	27 (11.1 %)
Presence of abscess (yes/no)	196 (80.3 %)	48 (19.8 %)
ICU (yes/no)	49 (20.1 %)	195 (79.9 %)
Operation (yes/no)	181 (74.2 %)	63 (25.8 %)

Supplement table S7. Continuous variables in RPE-negative patients.

Without RPE	Mean ± SD	Maximum	Median	Minimum
Age (years)	38.7 ± 19.6	88	37	0
CRP (mg/l)	92.5 ± 74.1	417	78	1
Hospital stay lenght (days)	3.36 ± 3.06	25	3	0
Maximal abscess diameter (if present, mm)	27.7 ± 14.1	115	27	7

Supplement table S8. Demographics and binary variables in RPE-negative patients.

Without RPE		
Sex (male/female)	126 (51.6 %)	109 (46.4 %)
Adult/Pediatric (yes/no)	205 (87.2 %)	30 (12.8 %)
Presence of abscess (yes/no)	138 (58.7 %)	97 (41.3 %)
ICU (yes/no)	7 (3.0 %)	228 (97.0 %)
Operation (yes/no)	148 (63.0 %)	87 (37.0 %)
